# Supplementary material for: Comparative multiomics analysis of cell physiological state after culture in a basket bioreactor
Source: Sci Rep. 2022 Nov 23;12:20161. doi: 10.1038/s41598-022-24687-4 (PMC9686226; doi:10.1038/s41598-022-24687-4)
Supplement: Supplementary file 1 — Supplementary Information 1. [file 41598_2022_24687_MOESM1_ESM.zip › raw data/Proteomic raw data/6.KEGGenrichment/EG.vs.CG/UP/pathway/src/03040.html]

map03040


- K12890, K12891, K12892, K12893, K12896, K21123, K12897, K12900

- Up regulated proteins


A0A0D9QYJ1(Description:Serine/arginine-rich splicing factor 2;Gene:SRSF2;log2FC:1.2387)

- K12860

- Up regulated proteins

A0A0D9RJ55(Description:Uncharacterized protein;Gene:CDC5L;log2FC:inf)

- K12878, K12879, K12880, K12881

- Up regulated proteins

A0A0D9RYG7(Description:Death domain-containing protein;Gene:THOC1;log2FC:1.3665)

Close
